# Immunoglobulin G4–Related Disease of the Sternoclavicular Joint: A Case Report

**DOI:** 10.1002/ccr3.70366

**Published:** 2025-04-01

**Authors:** Remi Mizuta, Shinnosuke Takemoto, Mutsumi Ozasa, Sawana Ono, Takahito Fukuda, Ryuta Tagawa, Fumiko Hayashi, Kazumasa Akagi, Hiromi Tomono, Hirokazu Taniguchi, Midori Matsuo, Hiroshi Gyotoku, Takahiro Takazono, Hiroshi Ishimoto, Noriho Sakamoto, Keitaro Matsumoto, Yasushi Obase, Tomoya Nishino, Hiroshi Mukae

**Affiliations:** ^1^ Department of Respiratory Medicine The Japanese Red Cross Nagasaki Genbaku Hospital Nagasaki Japan; ^2^ Department of Respiratory Medicine Nagasaki University Graduate School of Biomedical Sciences Nagasaki Japan; ^3^ Department of Respiratory Medicine Saiseikai Nagasaki Hospital Nagasaki Japan; ^4^ Department of Pathology Informatics Nagasaki University Graduate School of Biomedical Sciences Nagasaki Japan; ^5^ Clinical Oncology Center Nagasaki University Hospital Nagasaki Japan; ^6^ Clinical Research Center Nagasaki University Hospital Nagasaki Japan; ^7^ Department of Surgical Oncology Nagasaki University Graduate School of Biomedical Sciences Nagasaki Japan; ^8^ Department of Nephrology Nagasaki University Graduate School of Biomedical Sciences Nagasaki Japan

**Keywords:** bone, IgG4‐related disease, sternoclavicular joint, tumor

## Abstract

This case highlights the possibility that Immunoglobulin G4‐related disease (IgG4‐RD) lesions can also occur in the sternoclavicular joint. If a neoplastic lesion is found in the sternoclavicular joint, a biopsy should be attempted to diagnose IgG4‐RD as a differential.

## 1 Introduction

Immunoglobulin G4‐related disease (IgG4‐RD) is an increasingly recognized immune‐mediated condition comprising a group of disorders that share specific pathological, serological, and clinical features [1]. IgG4‐RD sometimes causes sclerosing pseudotumor formation in multiple organs [2]. Because of their infiltrating and non‐specific radiation findings, pseudotumors are often misidentified as true neoplasms, including sarcoma, bone metastasis, or infection, before biopsy. IgG4‐RD can manifest as a single‐ or multi‐organ disease and can include bone structures, such as the temporal bone [3] and maxillary sinus [4], with various presentations, diagnoses, and treatments. Bone IgG4‐RD has been previously described; however, data on IgG4‐RD in the sternoclavicular joint are lacking. To the best of our knowledge, there are no reports on the involvement of the sternoclavicular joint in IgG4‐RD. This is the first case report of IgG4‐RD in the sternoclavicular joint, with details of its clinical course, diagnostic challenges, and treatment of this rare illness.

### 1.1 Case History

**FIGURE 1:**
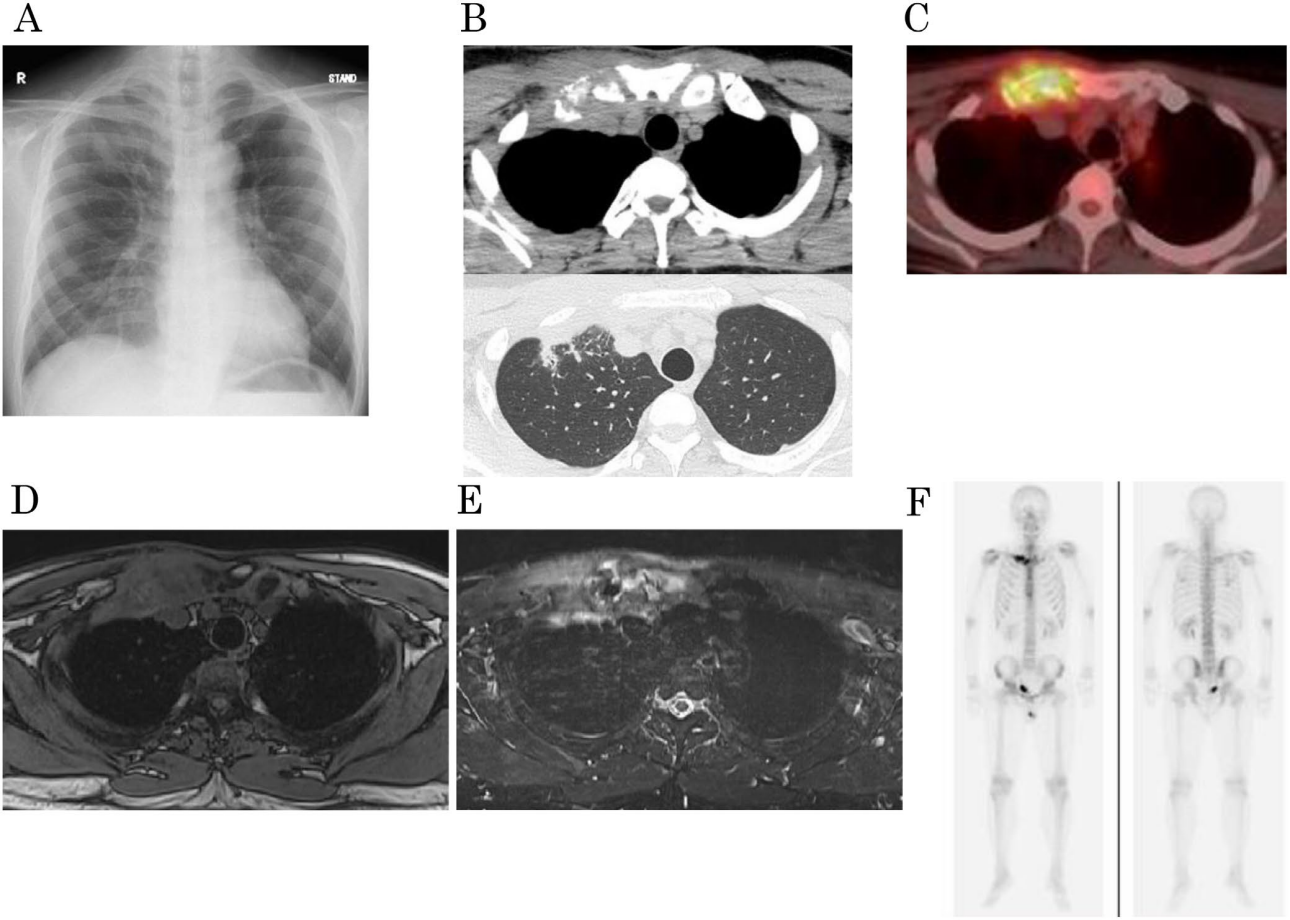
Radiographic examinations. (A) Chest X‐ray. (B) Chest computed tomography (CT). (C) Fluorodeoxyglucose positron emission CT. (D) Chest Magnetic Resonance Imaging (MRI) (T1‐weighted). (E) Chest MRI (T2‐weighted). (F) Techbetium‐99 m bone scintigraphy.

A 45‐year‐old man with a history of atopic dermatitis (AD), cataract surgery at the age of 16 years, sinus cyst surgery at the age of 31 years, no family history of note except that his brother had asthma, no smoking history, and no drinking habits. He was referred to our hospital with right shoulder pain. Chest radiography revealed pleural thickening at the lung apex (Figure 1A). Computed tomography (CT) revealed an osteolytic lesion in the right sternoclavicular joint, including the right first rib and right clavicle, along with thickening of the surrounding soft tissue and right pleura (Figure 1B).

### 1.2 Examination/Diagnosis

**FIGURE 2:**
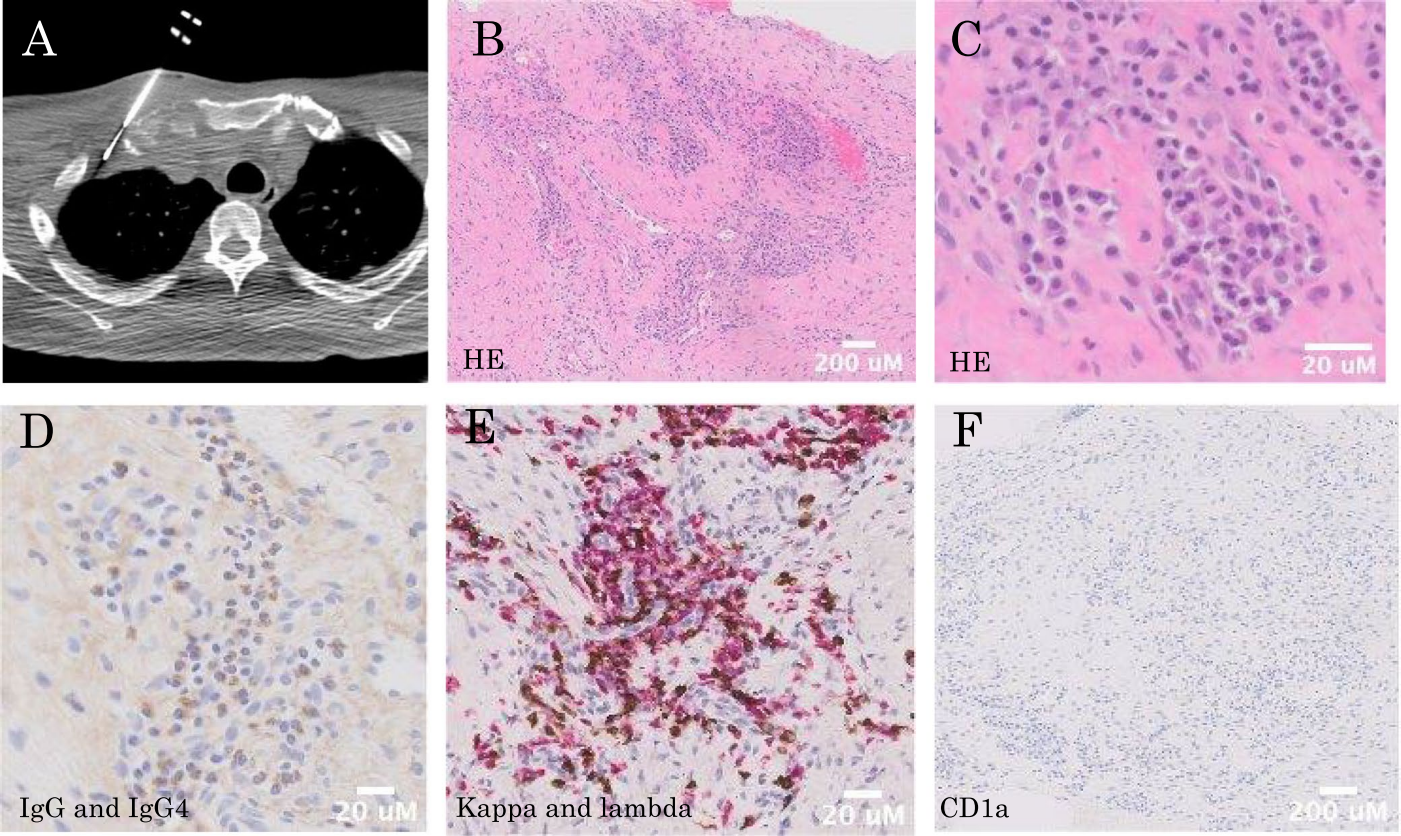
Histopathological and immunohistochemical findings of thickening of the right pleura. (A) CT‐guided needle biopsy (B) Hematoxylin and eosin (HE) staining (×10). (C) HE staining (×100). Plasma cell clusters were evident in the background of the fibrous components. (D) Immunohistochemical (IHC) staining for IgG (×100) (A57H, NCHIREI BIOSCIENCES) and IgG4 (×100) (HP6025, NCHIREI BIOSCIENCES). The IgG/IgG4 ratio was approximately 60%. (E) Kappa (red) and lambda (brown) light chain mRNA in situ hybridization (×100) (Kappa: BOND Kappa Probe, Leica. Lamda: BOND Lambda Probe, Leica). The kappa/lambda ratio was normal. (F) IHC staining for CD1a (×20) (MTB1; Leica). No detectable CD1a‐positive cells were observed.

**FIGURE 3:**
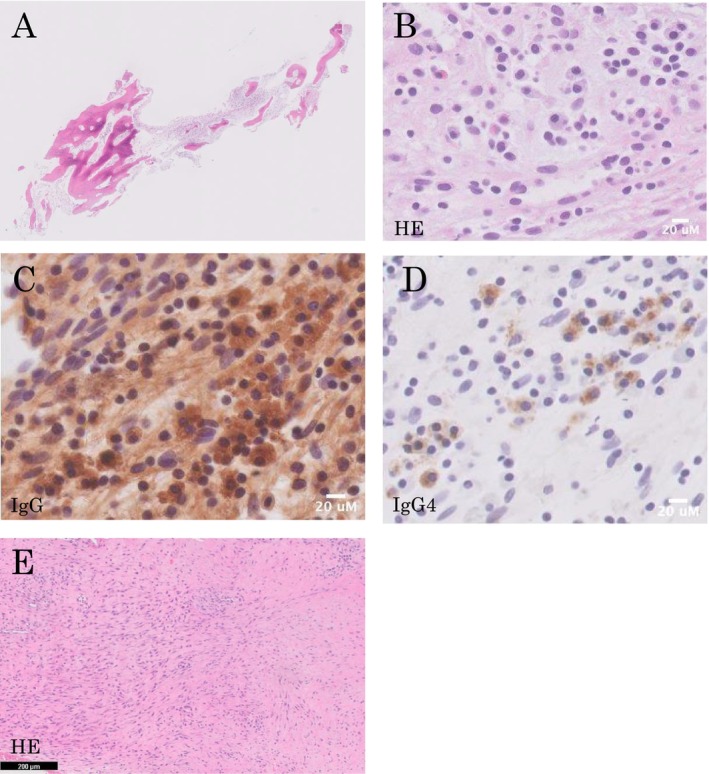
Histopathological and immunohistochemical findings of an osteolytic lesion in the right clavicle. (A) HE staining ×0.5. (B) HE staining (×100). Plasma cells were collected from around the clavicles. (C) IHC staining of IgG (×100). (D) IHC staining for IgG4 (×100). The IgG/IgG4 ratio is approximately 25%. The specimen contained 30 high‐power‐field IgG4‐positive cells. (E) HE staining (×200). This indicates the storiform fibrosis.

**FIGURE 4:**
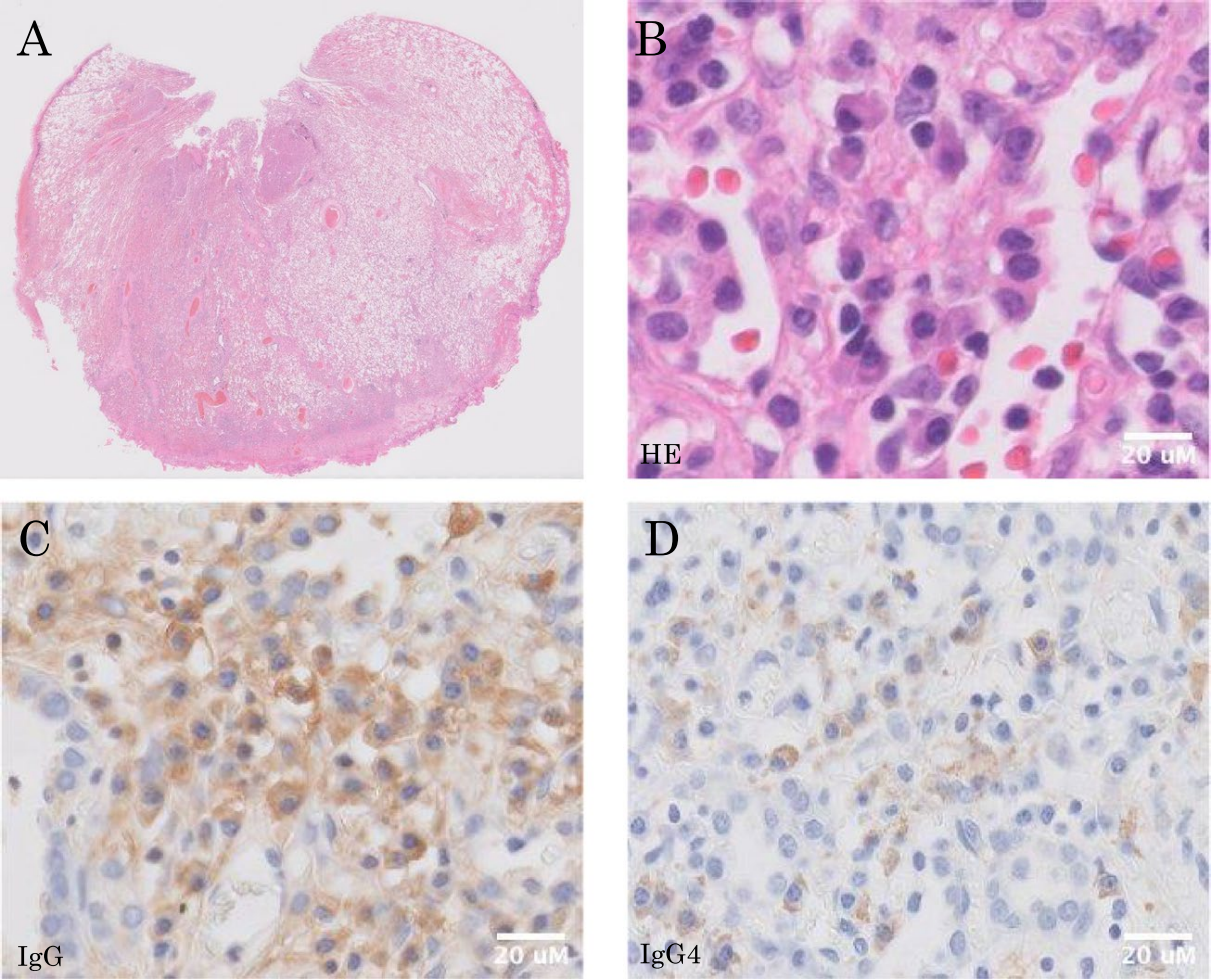
Histopathological and immunohistochemical findings of the lung. (A) HE staining ×0.5. (B) HE staining ×100. Plasma cells and histiocytes were collected around the bronchial blood vessels and pleura. (C) IHC staining of IgG (×100). (D) IHC staining for IgG4 (×100). An IgG/IgG4 ratio of approximately 30% is observed. The specimen showed 30/HPF IgG4‐positive cells.

A bronchoscopy and transbronchial biopsy of the right upper lobe was performed three times to diagnose right pleural thickening. However, we failed to obtain proper specimens. A CT‐guided needle biopsy (CTNB) of the right pleural thickening lesion was performed (Figure 2A). The specimen showed lymphoplasmacytic tissue infiltration, mainly IgG4‐positive plasma cells and lymphocytes, with granulation tissue (Figure 2B,C). Immunohistochemical (IHC) staining revealed IgG4‐positive plasma cells (30/high power field) and a high proportion of IgG4/IgG cells (60%) (Figure 2D). Other IHC staining showed a normal kappa/lambda ratio, and no CD1a‐positive cells, plasmacytoma, or Langerhans cell histiocytosis were ruled out (Figure 2E,F). Positron emission tomography/computed tomography (PET/CT) revealed increased metabolic activity in the right sternoclavicular joint and first rib (Figure 1C). Chest magnetic resonance imaging (MRI) revealed a low signal on T1‐weighted images and a high signal on T2‐weighted images of the right sternoclavicular joint (Figure 1D,E). Because the possibility of a malignant tumor could not be ruled out, a surgical biopsy was planned. Thoracoscopic partial resection of the right upper lung lobe, biopsy of the thickened right pleura, and biopsy of the osteolytic lesion in the right clavicle near the right sternoclavicular joint were performed. A specimen of the osteolytic lesion in the right clavicle showed a large number of lymphocytes around the clavicle. IHC staining revealed IgG4‐positive plasma cells (30/high power field) and a high proportion of IgG4/IgG cells (25%) (Figure 3). Although the IgG/IgG4 ratio was slightly lower, the lung and pleural specimens showed similar characteristics with no evidence of malignant cells, including plasmacytoma (Figures 3 and 4). The storiform fibrosis was found partially (Figure 3E). In addition to solid cancer, we considered sarcoidosis, malignant lymphoma, collagen disease, and infection due to immunodeficiency as diseases that could cause neoplastic lesions of the right sternoclavicular joint. However, no physical findings or laboratory workup results were observed, including those for soluble interleukin‐2 receptor (sIL2‐R), rheumatoid factor (RF), anti‐cyclic citrullinated peptide (CCP) antibody, matrix metalloproteinase‐3 (MMP3), anti‐SS‐A antibody, SS‐B antibody, antinuclear antibody (ANA), immunoglobulin G/A/M, and interferon gamma release assay, which were all normal (Table 1). Synovitis, acne, pustulosis, hyperostosis, and osteitis (SAPHO) syndrome is a disease‐causing symmetric bone lesion and differential disease [5]. Technetium‐99 m bone scintigraphy revealed an asymmetric accumulation of radioisotopes in the sternoclavicular joint (Figure 1F), and the possibility of SAPHO syndrome was considered low. Finally, IgG4‐RD was diagnosed based on the characteristic osteolytic lesion in the right sternoclavicular joint with pleural thickening on a chest CT, elevated serum IgG4 levels (631 mg/dL; Table 1), and the pathological findings described above.

**TABLE 1:** Laboratory testing.

Blood count		
White blood cell	9400	/μL
Neutrophil	78.7	%
Lymphocyte	13.9	%
Eosinophil	2.4	%
Hemoglobin	12	mg/dL
Platelet	41	×10^4/μL
Coagulation		
Prothrombin time‐international normalized ratio	1.1	
Activated partial thromboplastin time	33.1	sec
Tumor marker		
Carcinoembryonic antigen	1.1	g/dL
Cytokeratin19 fragment	1.4	g/dL
Pro‐gastrin‐releasing peptide	44.6	pg/dL
Soluble interleukin‐2 receptor	386	U/L
Biochemistry		
Total protein	8.3	g/dL
Albumin	3	g/dL
Total Bilirubin	0.4	mg/dL
Aspartate aminotransferase	13	U/L
Alanine aminotransferase	9	U/L
Alkaline phosphatase	178	U/L
Gamma‐glutamyl transpeptidase	17	U/L
Lactate dehydrogenase	142	U/L
Creatine kinase	68	U/L
Blood urea nitrogen	18	mg/dL
Creatinine	0.65	mg/dL
Sodium	142	mmol/L
Potassium	4	mmol/L
Chloride	106	mmol/L
Phosphorus	2.7	mg/dL
Calcium	8.9	mg/dL
C‐reactive protein	9.59	mg/dL
Erythrocyte sedimentation rate	93	mm/h
Serology		
Angiotensin‐converting enzyme	8.6	U/L
Immunoglobulin G	1920	mg/dL
Immunoglobulin A	350	mg/dL
Immunoglobulin M	40.7	mg/dL
Immunoglobulin E	4238	mg/dL
Immunoglobulin G4	631	mg/dL
50% hemolytic unit of complement	43.4	U/mL
Complement C3	133	U/mL
Complement C4	29.2	U/mL
Anti‐nuclear antibody	< 80 ×	
Rheumatoid factor	< 15	U/mL
Cyclic citrullinated peptide antibody	< 4.5	U/mL
Matrix metalloproteinase‐3	39.9	ng/mL
Sjogren syndrome‐A antibody	< 10	U/mL
Sjogren syndrome‐B antibody	< 7	U/mL
Thymus and activation‐regulated chemokine	755	pg/mL
Krebs von den lungen‐6	169	U/mL
Surfactant protein‐A	23.8	ng/mL
Surfactant protein‐D	31	ng/mL
Interferon gamma release assay	Negative	

### 1.3 Treatment/Outcome

**FIGURE 5:**
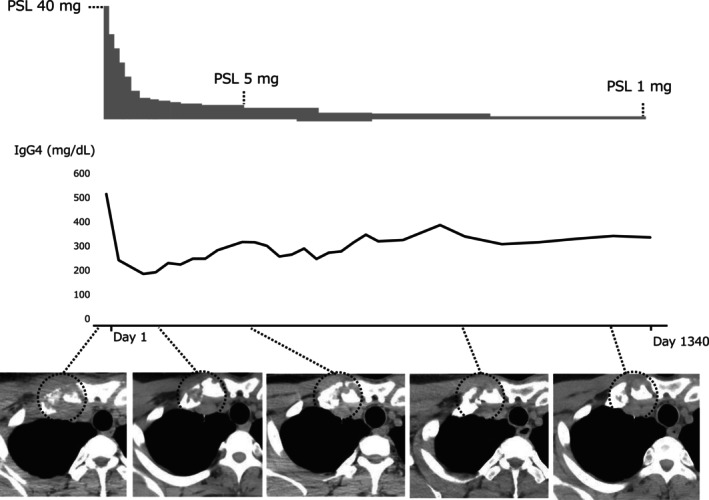
Time course of the case.

It was difficult to completely rule out diseases other than IgG‐RD. However, since IgG‐RD was the most likely cause, we treated with prednisolone in accordance with the treatment of IgG4‐RD [6]. The patient was started on 40 mg of prednisolone per day, and chest CT on Day 28 after treatment showed slight shrinkage of the osteolytic lesion in the right sternoclavicular joint and an obvious improvement in soft tissue thickening (Figure 5). Serum IgG4 levels were reduced (Figure 5). Although IgG4‐RD is considered definitive, malignant diseases, including carcinomas, sarcomas, and plasma cell tumors, have not been completely excluded. A follow‐up CT scan revealed that the osteolytic lesion in the right sternoclavicular joint increased every other month for 44 months. However, the osteolytic lesion has not increased; rather, it has shrunk (Figure 5). We wondered whether the current case was IgG4‐RD. So, we consulted a rheumatologist. The rheumatologist opined that IgG4‐RD was diagnosable if the malignancy had been denied. The absence of long‐term recurrence proves that it was not malignant and makes it more certain that it was IgG4‐RD.

## 2 Discussion

IgG4‐RD of the sternoclavicular joint is rare. In the current case, we diagnosed IgG4‐RD because the biopsy specimens showed a high IgG4/IgG ratio and many other diagnoses were excluded.

### 2.1 Differential Diagnosis

In the current case, differential diagnoses need to be ruled out. In addition to the differential diagnosis described above, mimickers of IgG4‐RD, including Rosai‐Dorfman disease (RDD) [7] and Erdheim‐Chester disease (ECD) [8], were considered. RDD and ECD are rare histiocytic disorders. The former is associated with massive lymphadenopathy and extranodal disease, whereas the latter is associated with multiple organ and tissue dysfunctions. Both of these factors can cause bone lesions. In the current case, no CD1a‐positive Langerhans cells were observed in the tissues (Figure 2E). Although strict differentiation is difficult, the ECD and RDD were considered negative. In the current case, the osteolytic lesion was suspected to be a malignant lesion, such as a sarcoma. The lesion could not be resected en bloc, and only a biopsy was performed. Ikeda et al. retrospectively analyzed 26 patients with IgG4‐RD, and two patients (7.7%) developed life‐threatening malignancies [9]. In the current case, the osteolytic lesion shrank slightly in response to prednisolone and did not increase in size for approximately a year. The osteolytic lesion was not malignant but related to IgG4‐RD, an immune‐related condition comprising a group of disorders that share specific pathological, serological, and clinical features [1].

### 2.2 Comparison With Other Cases

Pace et al. reported an IgG4‐related sclerosing disease of the maxillary sinus with bone destruction. Histology and IHC staining revealed numerous plasma cells, more than 20% of which were of the IgG4 type [4]. Vuncannon et al. reported an IgG4‐RD in the temporal bone. The CT imaging showed soft tissue opacification of the middle ear in the temporal bone, and pathology and IHC showed IgG4‐positive cells greater than 50/HPF, and the ratio of IgG4+/IgG+ plasma cells was 30% [3]. Lin et al. reported IgG4‐RD involving soft tissue adjacent to the thoracic vertebrae [10]. Molligan et al. reported 25 patients with tumor of the sternoclavicular joint and its pathological diagnosis, including five cases of cartilage, two cases of chondrosarcoma, one case of synovial chondrogenesis, one case of dense fibrous tissue, one case of spindle cell lesion with chondrogenic differentiation, one case of degenerative bone, one case of synovium plus fibrovascular tissue, one case of degenerative changes, one case of benign uncertain etiology, one case of synovial proliferation, and one case of hypocellular fibrocartilaginous lesion. IgG4‐RD was not included [11]. As mentioned above, this is the first case of IgG4‐RD involving the sternoclavicular joint. To compare with other cases of IgG4‐RD with bone lesions involving different anatomical sites, serum IgG4 was higher than previously reported [12, 13]. This was due to the fact that the patient originally had atopic dermatitis. Soluble IL2‐R was lower than in three cases of IgG4‐RD with skull base lesions [13]. IgG4‐RD with bone lesions may have different pathophysiology depending on its location.

### 2.3 Atopic Dermatitis and IgG4‐RD

However, the pathogenesis of IgG4‐RD remains unclear. Watanabe et al. reported that nine of 61 patients with AD had higher IgG4‐anti‐DFS antibodies and referred to the relationship between IgG4 and atopic dermatitis [14]. A possible mechanism was proposed that Th2 cytokines, important mediators of atopic disease such as interleukin (IL)‐4 and IL‐13, promote class conversion to IgG4 and induce IgG‐RD. Although no cytokines such as IL‐4 or IL‐13 were measured in this case, high blood Th2 cytokines might have suggested an association between IgG‐RD and AD [15]. AD might have affected the IgG‐RD in the current case.

### 2.4 Limitation of the Study

There were some limitations in this report. These findings were based on a single patient. The generalizability was limited because there were no patients harboring tumor‐like lesions in the sternoclavicular Joint.

## 3 Conclusions

To our knowledge, this is the first report of sternoclavicular joint involvement in a patient with IgG4‐RD. The pathogenesis is still poorly understood, and IgG4‐RD can result in lesions in any organ. Therefore, IgG4‐RD should be considered in the diagnosis of bone or joint lesions of unknown etiology.

## Author Contributions

**Remi Mizuta:** conceptualization, resources, writing – original draft. **Shinnosuke Takemoto:** conceptualization, data curation, supervision, validation, writing – review and editing. **Mutsumi Ozasa:** methodology, validation, writing – review and editing. **Sawana Ono:** writing – review and editing. **Takahito Fukuda:** writing – review and editing. **Ryuta Tagawa:** writing – review and editing. **Fumiko Hayashi:** writing – review and editing. **Kazumasa Akagi:** writing – review and editing. **Hiromi Tomono:** writing – review and editing. **Hirokazu Taniguchi:** writing – review and editing. **Midori Matsuo:** writing – review and editing. **Hiroshi Gyotoku:** writing – review and editing. **Takahiro Takazono:** writing – review and editing. **Hiroshi Ishimoto:** writing – review and editing. **Noriho Sakamoto:** writing – review and editing. **Keitaro Matsumoto:** writing – review and editing. **Yasushi Obase:** writing – review and editing. **Tomoya Nishino:** writing – review and editing. **Hiroshi Mukae:** project administration, writing – review and editing.

## Consent

Written informed consent was obtained from the patient to publish this report in accordance with the journal's patient consent policy.

## Conflicts of Interest

The authors declare no conflicts of interest.

## Data Availability

The data underlying this article will be shared on reasonable request to the corresponding author.
